# Mechanical Prophylaxis Management of Venous Thromboembolism in a General Surgical Setting: A Retrospective Cohort Study

**DOI:** 10.1002/hsr2.71888

**Published:** 2026-04-02

**Authors:** Lijia Wang, Chen Deng

**Affiliations:** ^1^ Nursing Department, The Third Xiangya Hospital Central South University Changsha Hunan China

**Keywords:** hepatectomy, mechanical prophylaxis, pancreatectomy, venous thromboembolism

## Abstract

**Background and Aims:**

Patients undergoing pancreatectomy and hepatectomy are at high risk of venous thromboembolism (VTE) and bleeding. Mechanical prophylaxis effectively prevents VTE, yet optimal management strategies remain debated. This study compared two mechanical prophylaxis regimens in these surgical populations.

**Methods:**

This retrospective study (January 2017–December 2022) included patients managed under distinct protocols: those admitted during 2017–2018 received the Assessment‐Prophylaxis (AP) regimen (*n* = 176), while those admitted during 2019–2022 received the Assessment‐Screening‐Prophylaxis‐Evaluation (ASPE) regimen (*n* = 627). Univariate analysis (chi‐square test) and multivariate logistic regression were performed to assess hospital‐associated VTE (HA‐VTE) outcomes.

**Results:**

The ASPE group demonstrated higher VTE risk assessment rates at admission (94.4% vs. 46.0%, *p* < 0.001), ultrasound screening rates (45.3% vs. 1.2%, *p* < 0.001), and postoperative mechanical prophylaxis rates (98.4% vs. 92.8%, *p* = 0.001). However, HA‐VTE incidence did not differ significantly between groups (1.9% vs. 1.1%, *p* = 0.486). Multivariate analysis identified prolonged hospitalization (OR = 1.057, 95% CI: 1.024–1.090, *p* = 0.001) and admission VTE risk assessment (OR = 9.347, 95% CI: 1.089–80.214, *p* = 0.042) as independent risk factors for HA‐VTE, while postoperative mechanical prophylaxis reduced HA‐VTE risk (OR = 0.081, 95% CI: 0.016–0.408, *p* = 0.002).

**Conclusions:**

The ASPE regimen improved compliance with VTE prevention protocols. Postoperative mechanical prophylaxis significantly reduced HA‐VTE incidence, supporting its clinical adoption.

## 1 Background

Venous thromboembolism (VTE), which includes deep vein thrombosis (DVT) and pulmonary thromboembolism (PTE), presents significant challenges due to its high incidence, mortality rate, and complexities in diagnosis and management. VTE may have an insidious onset and nonspecific symptoms, contributing to unanticipated and perioperative deaths in hospital settings [1]. In Europe and the United States, the incidence of VTE is estimated to range from 1 to 2 per 1000 person‐years [2]. Data from approximately 100,000 patients across 26 countries revealed a 30‐day mortality rate of 2.6% for distal DVT, 3.3% for proximal DVT, and 5.2% for pulmonary embolism [3]. Survey data from 2016 to 2020 from 4480 hospitals in China indicates a concerning trend, showing a surge in the population's incidence of VTE from 3.26 to 8.53 per 10,000 individuals. Similarly, HA‐VTE increased from 0.48‰ to 0.56‰ during the same period. Additionally, the case fatality rate of acquired VTE in hospitals rose from 5.64% to 6.18% from 2016 to 2020 [4]. The treatment of VTE can be substantially more expensive than its prevention [5], and standardized and rational VTE prophylaxis measures can reduce VTE incidence by 50% to 60% [6]. However, a large study comprising over 100,000 hospital admissions revealed that only 15% of patients categorized as “high risk for VTE” received appropriate prophylaxis [7]. In China, only 9.3% of surgical patients are given reasonable preventive measures [8].

Numerous studies indicate that intermittent pneumatic compression (IPC) can reduce postoperative DVT risk without increasing bleeding hazards. Thus, IPC could be a viable alternative to pharmacologic thromboprophylaxis, especially in surgical settings [9]. In the general pancreatectomy patient population, venous thromboembolism (VTE) incidence is estimated at 2%–4%, while post‐pancreatectomy hemorrhage (PPH) occurs at 6%–8%. Notably, PPH incidence is twice that of VTE and carries substantially higher mortality, ranging from 22% to 47% [10]. PPH is classified as early‐onset (within 24 h postoperatively) or late‐onset (> 24 h postoperatively). Early PPH typically results from technical failures of intraoperative hemostasis or perioperative coagulopathy. Late PPH, which often manifests days or weeks later, usually stems from surgical complications such as: Enzymatic digestion of vascular walls by pancreatic exocrine enzymes (e.g., trypsin) secondary to pancreatic leakage; Intra‐abdominal infection involving peri‐pancreatic vasculature; or pseudoaneurysm formation due to vascular injury during resection [11]. Critically, late‐onset PPH portends a worse prognosis than early PPH, indicating that pancreatectomy patients remain at high bleeding risk for an extended duration [12]. Evidence indicates that the risk of VTE following hepatectomy increases proportionally with the extent of liver resection [13]. According to the American College of Surgeons' National Surgical Quality Improvement Program (NSQIP) analysis of 5651 patients, symptomatic VTE occurred postoperatively in 2.9% of liver surgery cases, with the highest incidence observed after major resections [14]. Concurrently, postoperative intra‐abdominal hemorrhage—a leading cause of morbidity and mortality after hepatectomy—occurs in 1%–5% of cases [15]. Notably, coagulation dysfunction may develop postoperatively even in patients with normal preoperative coagulation profiles and liver function tests. Contributing factors include massive blood transfusion, underlying liver disease, significant intraoperative blood loss, dilutional coagulopathy from fluid resuscitation, resection extent, and transient synthetic dysfunction of the remnant liver. Importantly, these coagulopathic changes persist throughout the postoperative period [16].

When bleeding risk is deemed very high or the consequences of major hemorrhage are potentially catastrophic—mechanical prophylaxis with IPC is recommended [17]. Therefore, mechanical prophylaxis alone was implemented in this study, with pharmacological prophylaxis deliberately omitted. This retrospective cohort study specifically compares two distinct mechanical thromboprophylaxis modalities and evaluating their comparative effectiveness in VTE prevention within this surgical population.

## 2 Methods

### 2.1 Design

This STROBE‐compliant retrospective cohort study compared two mechanical prophylaxis protocols: the AP regimen (basic risk assessment and prophylaxis) and the ASPE regimen (integrated assessment, screening, prophylaxis, and evaluation).

### 2.2 Study Setting and Sample

**Figure 1:**
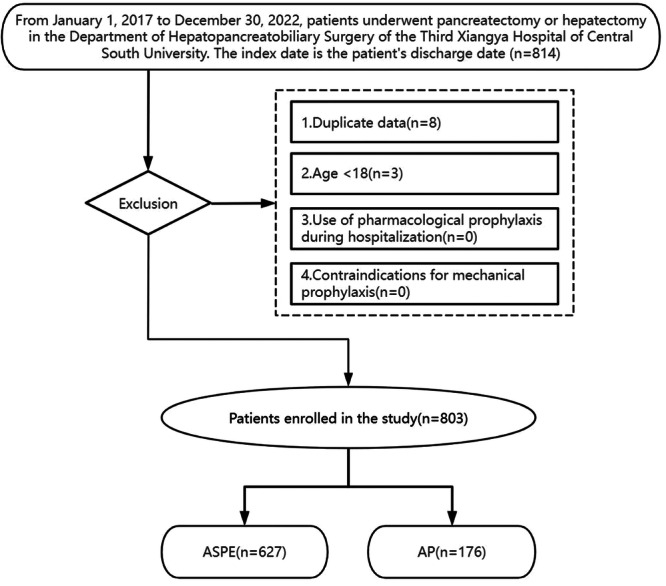
Flow chart of the selection of 803 patients in this study. AP, Assessment‐Prophylaxis; ASPE, Assessment‐Screening‐Prophylaxis‐Evaluation.

Our study was conducted in the Department of Hepatopancreat Obiliary Surgery, Third Xiangya Hospital of Central South University. This study enrolled patients who underwent mechanical preventive management for VTE in the Department of Hepatopancreat Obiliary Surgery at the Third Xiangya Hospital of Central South University from 2017 to 2022. The inclusion criteria were: (1) age ≥ 18 years, (2) voluntary participation with signed informed consent, (3) undergoing pancreatectomy or hepatectomy during hospitalization, and (4) absence of contraindications for mechanical prophylaxis. Exclusion criteria comprised the use of pharmacological prophylaxis during hospitalization. Two distinct protocols were implemented: the Assessment‐Prophylaxis protocol (AP) for patients admitted during 2017–2018 (*n* = 176), and the Assessment‐Screening‐Prophylaxis‐Evaluation protocol (ASPE) for those admitted during 2019–2022 (*n* = 627) (Figure 1).

### 2.3 Data Collection

Clinical data were collected from the hospital database, which included patient baseline data such as gender, age, length of stay, type of operation, and Caprini score. The primary outcome was HA‐VTE incidence. Secondary outcomes included VTE risk assessment rates, ultrasound screening rates, mechanical prophylaxis rates.

### 2.4 Procedures

**Figure 2:**
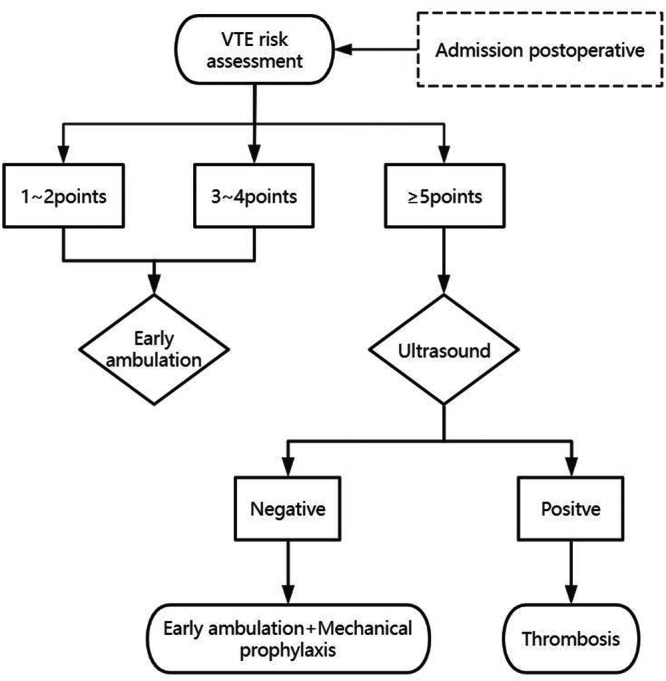
Flow of the AP regimen.

The following measures were implemented for the AP group (Figure 2): (1) VTE risk was assessed by nurses upon admission to the hospital and after surgery. (2) Patients with a Caprini score equal to or greater than 5 points received ultrasound examinations. Subsequently, patients with negative results received mechanical prophylaxis, while those with positive results received thrombus treatment. (3) Early mobilization protocols comprised two key interventions: postoperative ambulation initiation was actively encouraged within the earliest feasible timeframe and foot‐ankle exercise was systematically performed during bed confinement periods (with the same protocol as the ASPE group). Figure 2 illustrates the flow of the AP regimen.

**Figure 3:**
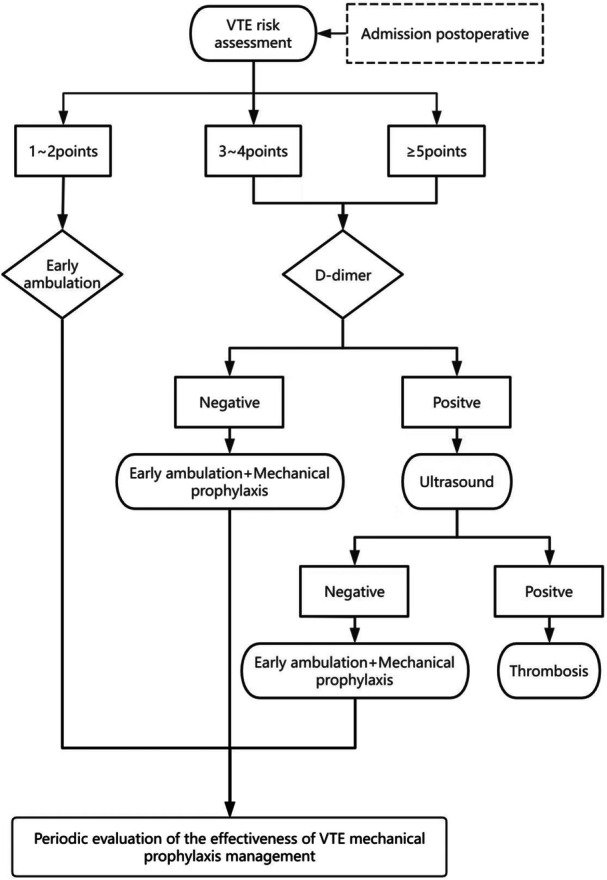
Flow diagram of the ASPE regimen.

The following measures were implemented for the ASPE regimen, as show in Figure 3:

1. A VTE management team was established, comprising the department director, head nurse, VTE management team leader, doctors, and nurses.
2. Assessment: Nurses were tasked with VTE risk assessment utilizing the Caprini score and patient education within 24 h of admission and 24 h following surgery.
3. Screening: VTE screening was conducted daily by designated nurses. d‐dimer testing was utilized to guide decisions on referral for ultrasonography in cases of suspected DVT. Nurses compiled a daily list of patients requiring ultrasonography to inform doctors.
4. Prophylaxis: Patients with a Caprini score of 0–2 received basic preventive measures, while those with a Caprini score of ≥ 3 received both basic preventive measures and mechanical preventive measures (with no confirmed contraindications). The basic preventive measures primarily consisted of early ambulation and foot‐ankle exercises, while mechanical preventive measures involved the use of IPC.
  a. Foot‐ankle exercise
    Patients performed slow and even ankle joint movements from the neutral position, including dorsiflexion, varus, plantar flexion, and valgus, with a range of activity of 20 degrees dorsiflexion, 30 degrees varus and valgus, and 40 degrees plantar flexion. Each movement was held for 3 s, with 20–30 sets per session, 10–15 times daily. Patients discontinued foot‐ankle exercises once they regained full mobility post‐surgery [18, 19].
  b. IPC
  Lower limb skin hygiene, skin temperature, blood transport, dorsal foot artery pulsation, and limb sensation were assessed daily before IPC.
  Monthly checks were conducted to ensure IPC functional status, performance, structural quality, and battery‐related functions.
  The size and length of mechanical devices were determined based on the patient's height and leg length, covering the lower limb from the lower leg to the thigh [20].
  A pressure of 35–40 mmHg was applied to the thigh and/or lower leg, with the leg cover inflated for approximately 10 s per cycle, followed by relaxation for 1 min before repeating the cycle [21].
  IPC was maintained during hospitalization until the patient achieved independent ambulation.
  A 60‐min daily IPC protocol was implemented, justified by the following evidence: intermittent pneumatic compression (IPC) exerts antithrombotic effects through dual mechanisms: fibrinolytic activation and hemodynamic enhancement. Evidence indicates that 1‐h IPC interventions significantly improve key fibrinolytic markers (e.g., ↑tPA activity, ↓PAI‐1, shortened euglobulin lysis time), with therapeutic efficacy exhibiting a time‐dependent relationship [22]. Current guidelines recommend extended application (≥ 18 h daily) for optimal prophylaxis. However, two critical constraints necessitated protocol modification in our study:resource limitations(inadequate IPC device availability) and patient compliance challenges (treatment intolerance due to extended wear) [8]. Consequently, a 1‐h IPC regimen was implemented to balance clinical feasibility with biologically significant fibrinolytic activation. This approach aligns with studies demonstrating that short‐duration IPC retains measurable antithrombotic effects.
5. Evaluation: The VTE management team leader was responsible for collecting monthly statistics on VTE risk assessment rate, mechanical prophylaxis rate, and the incidence of HA‐VTE. These data were analyzed, and meetings were organized to discuss corrective measures, aiming to continuously enhance VTE prevention and management.

### 2.5 Data Analysis

Data analysis was conducted using the SPSS software version 26. The chi‐square test was used to analyze categorical data and Student's *t*‐test was used to analyze continuous variables. Binary logistic regression was performed to assess the impact of various factors on the incidence of HA‐VTE. A *p*‐value < 0.05 was considered statistically significant, and two‐sided *p*‐values were reported.

## 3 Results

### 3.1 Participant Characteristics

Table 1 presents the demographics and characteristics of participants. The ASPE group comprised 627 patients, including 341 males (54.4%) and 286 females (45.6%). In terms of age distribution, 406 patients (64.8%) were aged 18–60 years, and 221 were aged over 60 years. Regarding BMI, 507 patients (80.9%) had a BMI ≤ 25, while 120 patients (19.1%) had a BMI > 25. The average length of stay was 20 days. In addition, 233 patients (37.2%) underwent pancreatectomy, and 394 patients (62.8%) underwent hepatectomy. On admission, 154 patients (21.6%) were at medium‐high risk of VTE, increasing to 619 patients (98.3%) post‐surgery. Among the patients, 453 cases (76.5%) had a Caprini score of 0–2 before the operation, while 139 cases (23.5%) had a Caprini score of 3 or higher before the operation. There were 11 cases (1.9%) with Caprini score ranging from 0 to 2 after the operation, and 563 cases (98.1%) with a Caprini score equal to or greater than 3 after the operation. The median d‐dimer level of the patients before the operation was 0.52 (0.01–11.03) mg/L, while that of the patients after the operation was 5.03 (0.15–23.07) mg/L.

**Table 1:** Baseline characteristics of the investigated study cohort.

Characteristics	ASPE (*n* = 627)	AP (*n* = 176)	*χ* ^2^/*T*	*p* value
Gender (%)				
Male	341 (54.4%)	89 (50.6%)	0.805	0.370
Female	286 (45.6%)	87 (49.4%)		
Age(years)			2.887	0.409
≤ 60	406 (64.8%)	108 (61.4%)	0.685	0.408
> 61	221 (35.2%)	68 (38.6%)		
BMI			0.644	0.422
≤ 25	507 (80.9%)	147 (83.5%)		
> 25	120 (19.1%)	29 (16.5%)		
Length of stay	20 (3–92)	19 (5–69)	0.553	0.580
Type of operation			5.907	0.015
Pancreatectomy	233 (37.2%)	48 (27.3%)		
Hepatectomy	394 (62.8%)	128 (72.7%)		
Caprini score (admission)			2.250	0.134
0–2	453 (76.5%)	68 (84.0%)		
≥ 3	139 (23.5%)	13 (16.0%)		
Caprini score (postoperation)			2.449	0.118
0–2	11 (1.9%)	4 (4.6%)		
≥ 3	563 (98.1%)	83 (95.4%)		
d‐dimer (admission)	0.52 (0.01–11.03)	0.86 (0.04–7.95)	−0.860	0.390
d‐dimer (postoperation)	5.03 (0.15–23.07)	5.03 (0.64–30.72)	−1.720	0.086

Abbreviations: AP, Assessment‐Prophylaxis; ASPE, Assessment‐Screening‐Prophylaxis‐Evaluation; VTE, venous thromboembolism.

The AP group comprised 176 patients, including 89 males (50.6%) and 87 females (49.4%). A total of 108 patients (61.4%%) were aged 18 to 60 years, and 68 patients (38.6%) were aged over 60 years. In terms of BMI, 147 patients (83.5%) had a BMI ≤ 25, while 29 patients (16.5%) had a BMI > 25. The median length of hospital stay was 19 days (5–69 d). Overall, 48 patients (27.3%) underwent pancreatectomy, 128 patients (72.7%) underwent hepatectomy. Among the patients, 68 cases (84.0%) had a Caprini score of 0–2 before the operation, while 13 cases (16.0%) had a Caprini score of 3 or higher before the operation. There were 4 cases (4.6%) with Caprini score ranging from 0 to 2 after the operation, and 83 cases (95.4%) with Caprini score equal to or greater than 3 after the operation. The median d‐dimer level of the patients before the operation was 0.86 (0.04–7.05) mg/L, while that of the patients after the operation was 5.03 (0.64–30.72) mg/L.

No statistically significant differences were observed in gender, age, BMI, length of stay, Caprini score, d‐dimer between the two groups (*p* > 0.05). However, statistically significant differences were found in the type of surgery between the two groups (*p* = 0.015).

### 3.2 Study Outcomes

Table 2 indicates a statistically significant difference in the VTE risk assessment rate at admission (rate difference: 0.48, 95% CI: 0.41, 0.55, *p* < 0.001) between the two groups. Similarly, a statistically significant difference in the VTE risk assessment rate was observed between the two groups after surgery (rate difference: 0.42, 95% CI: 0.34, 0.49, *p* < 0.001).

**Table 2:** Comparison of VTE risk assessment rate between the two groups.

Variables	ASPE (*n* = 627)	AP (*n* = 176)	Between‐group rate difference (95% CI)	*χ* ^2^	*p* value
VTE risk assessment rate (admission)	592 (94.4%)	81 (46.0%)	0.48 (0.41,0.55)	237.214	< 0.001
95% CI	(0.93,0.96)	(0.38,0.53)			
VTE risk assessment rate (postoperation)	574 (91.5%)	87 (49.4%)	0.42 (0.34,0.49)	167.450	< 0.001
95% CI	(0.89,0.93)	(0.42,0.57)			
**χ** ^**2**^	3.960	0.410			
***p*** **value**	0.047	0.522			

Abbreviations: AP, Assessment‐ Prophylaxis; ASPE, Assessment‐Screening‐Prophylaxis‐Evaluation; VTE, venous thromboembolism.

Further analysis revealed a statistically significant difference in the VTE risk assessment rate between admission and post‐operation in the ASPE group (*χ* ^2^ = 3.960, *p* = 0.047). However, no statistical significance was observed in the VTE risk assessment rate between admission and post‐operation in the AP group (*χ* ^2^ = 0.410, *p* = 0.522).

Table 3 indicates a statistically significant difference in the detection rate of venous color ultrasound between the two groups (rate difference: 0.44, 95% CI: 0.39, 0.49, *p* < 0.001).

**Table 3:** Comparison of ultrasound screening rate within the two groups.

Variables	ASPE (*n* = 563)	AP (*n* = 83)	Between‐group rate difference (95% CI)	*χ* ^2^	*p* value
Ultrasound screening rate	255 (45.3%)	1 (1.2%)	0.44 (0.39,0.49)	58.771	< 0.001
95% CI	(0.41,0.49)	(0.00,0.07)			

Abbreviations: AP, Assessment‐Prophylaxis; ASPE, Assessment‐Screening‐Prophylaxis‐Evaluation.

Table 4 illustrates no statistically significant difference in the mechanical prophylaxis rate at admission between the two groups (rate difference: −0.07, 95% CI: −0.26, 0.13, *p* = 0.421). However, a significant difference in the mechanical prophylaxis rate was observed after the surgery between the two groups (rate difference: 0.05, 95% CI: 0.00, 0.13, *p* = 0.01). The mechanical prophylaxis rate at admission and after the operation showed significant differences in the ASPE group (*χ* ^2^ = 575.11, *p* < 0.001), as well as in the AP group (*χ* ^2^ = 46.190, *p* < 0.001).

**Table 4:** Comparison of mechanical prophylaxis rate of the two groups.

Variables	ASPE	AP	Between‐group rate difference (95% CI)	*χ* ^2^	*p* value
Mechanical prophylaxis rate (admission)	12(8.6%)	2(15.4%)	−0.07(−0.26,0.13)	0.648	0.421
95%CI	(0.05,0.15)	(0.018,0.45)			
Mechanical prophylaxis rate (postoperation)	554(98.4%)	77(92.8%)	0.05 (0.00,0.13)	10.110	0.001
95%CI	(0.97,0.99)	(0.87,0.98)			
**χ** ^**2**^	575.11	46.190			
***P*** **value**	0.000	0.000			

Abbreviations: AP, Assessment‐Prophylaxis; ASPE, Assessment‐Screening‐Prophylaxis‐Evaluation.

Data analysis showed no statistical significance in the Incidence of HA‐ VTE (rate difference: 0.01, 95% CI: −0.020,0.02, *p* = 0.486) between the two groups (Table 5).

**Table 5:** Comparison of Incidence of HA‐ VTE between the two groups.

Variables	ASPE (*n* = 627)	AP (*n* = 176)	Between‐group rate difference (95% CI)	*χ* ^2^	*p* value
Incidence of HA‐ VTE	12 (1.9%)	2 (1.1%)	0.01 (−0.02,0.02)	0.485	0.486
95% CI	(0.01, 0.03)	(0.00, 0.04)			

Abbreviations: AP, Assessment‐Prophylaxis; ASPE, Assessment‐Screening‐Prophylaxis‐Evaluation.

### 3.3 Binary Logistic Regression

Binary logistic regression was conducted to evaluate the impact of various factors on the incidence of hospital‐associated VTE. The independent variables included gender, age, BMI, length of stay, type of operation, Caprini score (admission and postoperative), d‐dimer values (admission and postoperation), ultrasound screening rate, and mechanical prophylaxis rate (admission and postoperation). The incidence of HA‐VTE of the two groups served as the dependent variable; the independent variables are detailed in Table 6.

**Table 6:** Argument assignment descriptions.

Independent variables	Categories
Gender	0 = Male, 1 = Female
Age	0 = 60 or less 1 = greater than 60
BMI	0 = 25 or less 1 = greater than 25
Type of operation	0 = hepatectomy 1 = pancreatectomy
VTE risk assessment (admission)	No = 0, Yes = 1
VTE risk assessment (postoperative)	No = 0, Yes = 1
mechanical prophylaxis (postoperative)	No = 0, Yes = 1
Hospital‐associated VTE	No = 0, Yes = 1

**Figure 4:**
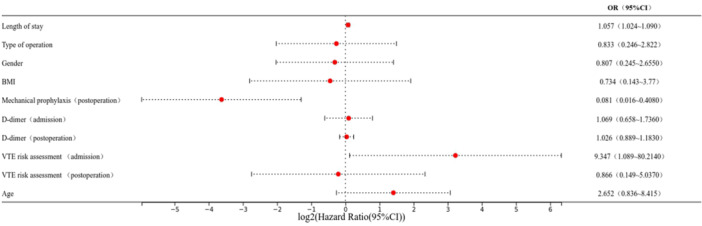
Risk factors of the two groups analyzed using binary logistic regression.

Figure 4 illustrates the results of a multivariable regression model, which was used to adjust for significant baseline characteristics. The results show that length of stay, VTE risk assessment at admission and the mechanical prophylaxis rate (postoperation) were identified as independent risk factors for HA‐ VTE in both groups (*p* < 0.05).

## 4 Discussion

All general surgery patients should undergo VTE risk assessment. Effective VTE prevention programs can be implemented based on accurate risk assessment to minimize the incidence of VTE. Based on clinical experience and a substantial amount of evidence‐based medical evidence, the Caprini risk assessment model (Caprini RAM) is recommended for assessing the risk of VTE in general surgery patients [23]. The Caprini RAM is a dynamic tool requiring ongoing patient evaluation during hospitalization and postoperative recovery. Changes in clinical status may prompt score adjustments, leading to modified treatment recommendations [24]. Thus, utilizing the Caprini scale for VTE risk assessment is a crucial initial step in VTE prevention and management. A multicenter cross‐sectional study in China revealed that only 9% of 13,609 hospitalized patients underwent VTE risk assessment as per the 9th edition of ACCP guidelines, with rates of 6.0% in medicine and 11.8% in surgery [25]. In this study, the VTE risk assessment rate at admission and post‐operation in the ASPE group surpassed that of the AP group, suggesting that enhancing mechanical prophylaxis management for VTE might effectively elevate the VTE risk assessment rate. These findings align with several contemporary studies reporting VTE risk assessment rates between 61.2% and 96.9% [26, 27, 28]. Paradoxically, admission VTE assessment correlated with higher HA‐VTE incidence, likely reflecting selection bias toward higher‐risk patients. Furthermore, the postoperative VTE risk assessment rate in the ASPE group was lower compared to the admission rate, indicating the need to improve the postoperative VTE risk assessment. The VTE risk assessment rate for hospitalized patients should be maintained at over 90% [29]. In this study, VTE risk assessment was conducted at admission and within 24 h post‐surgery. However, such assessments should also be repeated after procedures, transfers, changes in condition, and discharge. Therefore, the prevention program should be continuously adjusted based on these evaluations. Additionally, this study did not calculate the accuracy of VTE risk assessment, and future studies should explore this aspect to improve VTE prevention management. Ultrasound is the first investigation for VTE diagnosis due to its sensitivity, accuracy, and non‐invasive nature [30]. In this study, the rate of ultrasound screening in the ASPE group was significantly higher than in the AP group, suggesting that the implementation of ASPE VTE mechanical prophylaxis measures improved ultrasound screening. This finding aligns with previous studies reporting ultrasound screening rates between 19.8% and 30.7% in surgical patients [30, 31].

This study revealed that patients undergoing pancreatectomy and hepatectomy were at a high risk of bleeding. Therefore, mechanical prophylaxis was considered the most suitable method for these patients. The postoperative mechanical prophylaxis rate in the ASPE group was higher than in the AP group. No significant difference in mechanical prophylaxis rate was found between the two groups at admission, with both rates being relatively low. This suggests that the mechanical prophylaxis management program can potentially improve the implementation rate of mechanical prophylaxis. This finding is consistent with Peng's research [26]. However, in both groups, the mechanical prophylaxis rate at admission was remarkably lower than after the surgery. This difference could be attributed to the fact that high‐risk patients with VTE have better mobility before surgery and are encouraged to engage in activities to prevent thrombosis. This finding contrasts with Shrikhande and Zhang's study [30, 32]. Additionally, there is a lack of attention to preoperative patients who require mechanical prophylaxis, leading to the omission of mechanical prophylaxis [33], and low compliance. Therefore, preoperative VTE prophylaxis should be emphasized.

The **χ** ^**2**^ analysis in this cohort revealed no statistically significant intergroup difference in hospital‐acquired venous thromboembolism (HA‐VTE) incidence (*p* = 0.486), aligning with prior investigations [26, 34, 35, 36, 37, 38]. This apparent null association likely stems from two methodological considerations: the **χ** ^**2**^ test quantified crude between‐group differences without controlling for clinically relevant covariates, particularly hospitalization duration and VTE risk assessment at admission. Notably, subsequent multivariable logistic regression accounting for these parameters demonstrated mechanical prophylaxis as an independent protective factor (OR = 0.081, 95%CI = 0.016–0.408). With only 14 documented thrombotic events, the **χ**² test's capacity to detect modest therapeutic effects was inherently constrained.

### 4.1 Strengths and Limitations of the Study

First, as a single‐center retrospective cohort study with a restricted sample size (*N* = 803), the design inherently limits generalizability beyond similar tertiary care settings. Second, this study was a retrospective cohort study and the causal relationship could not be determined. Third, the absence of standardized metrics for patient‐initiated mobilization behaviors (e.g., frequency/duration of ambulation, compliance with ankle pump exercises) represents a critical methodological gap. In the context of Enhanced Recovery After Surgery (ERAS) protocols, the ASPE group potentially exhibited greater adherence to early mobilization regimens that may have introduced residual channeling bias favoring enhanced thromboprophylaxis outcomes.

## 5 Conclusions

Mechanical prophylaxis plays a crucial role in preventing VTE among surgical patients, offering a means to reduce VTE incidence without increasing bleeding risk. In this study, the ASPE regimen significantly increased the VTE risk assessment rate, ultrasound screening rate, and implementation rate of mechanical prophylaxis in patients undergoing pancreatectomy and hepatectomy. The results of Logistic regression analysis showed that the implementation of mechanical prophylaxis after surgery could effectively reduce the incidence of HA‐VTE. We have reasons to believe that the implementation of the new policy is effective in reducing HA‐VTE.

## Author Contributions

**Lijia Wang:** writing – original draft, writing – review and editing, software, formal analysis, investigation, visualization, data curation. **Chen Deng:** conceptualization, supervision, methodology, resources, project administration.

## Disclosure

The corresponding author Chen Deng affirms that this manuscript is an honest, accurate, and transparent account of the study being reported; that no important aspects of the study have been omitted; and that any discrepancies from the study as planned (and, if relevant, registered) have been explained.

## Ethics Statement

The Institutional Review Committee of the Third Xiangya Hospital of Central South University approved the study with registration number 24343. The manuscript is in line with STROBE guidelines. Given its non‐interventional, retrospective design and only focus on adverse events related to VTE data, informed consent was waived by the board.

## Conflicts of Interest

The authors declare no conflicts of interest.

## Data Availability

The data that support the findings of this study are available from the corresponding author upon reasonable request.
